# Effect of mitochondrial dysfunction on scar formation after spinal cord injury

**DOI:** 10.3389/fneur.2026.1782114

**Published:** 2026-04-30

**Authors:** Yuchen Zhang, Zhengxin Jin, Bin Ning

**Affiliations:** 1 Central Hospital Affiliated to Shandong First Medical University, Shandong First Medical University, Shandong Academy of Medical Sciences, Jinan, Shandong, China; 2 Jinan Central Hospital, Shandong University, Jinan, Shandong, China

**Keywords:** fibrotic scar, glial scar, mitochondrial dynamics, mitochondrial dysfunction, oxidative stress, spinal cord injury

## Abstract

Spinal cord injury (SCI) triggers a cascade of primary and secondary pathological events that culminate in the formation of glial and fibrotic scars, which constitute a major barrier to axonal regeneration and functional recovery. Emerging evidence highlights mitochondrial dysfunction as a central driver of this process. Mitochondria are essential for sustaining ATP production, maintaining redox balance, and regulating calcium homeostasis. Following SCI, direct mechanical disruption, oxidative stress, and calcium overload impair mitochondrial integrity, leading to energy metabolism collapse, excessive reactive oxygen species (ROS) accumulation, and disrupted mitochondrial dynamics. These alterations promote reactive gliosis, fibroblast activation, and maladaptive extracellular matrix deposition. Furthermore, defective mitophagy amplifies neuroinflammation and glial scar consolidation through the PINK1/Parkin and BNIP3/NIX pathways. Recent advances in mitochondrial-targeted therapies—including antioxidants (MitoQ, SS-31), metabolic modulators (AMPK agonists, NAD^+^ precursors), and strategies enhancing fusion or mitophagy—have demonstrated promising results in reducing scar formation and promoting neural repair. In addition, cutting-edge approaches such as mitochondrial transplantation, stem cell-derived mitochondrial transfer, and CRISPR-based mitochondrial gene editing provide new opportunities for restoring mitochondrial homeostasis. This review summarizes the multifaceted roles of mitochondrial dysfunction in SCI-induced scar formation and discusses novel therapeutic strategies targeting mitochondrial metabolism and dynamics to enhance neural regeneration.

## 1 Introduction

SCI, a serious injury to the central nervous system (CNS), frequently leads to significant functional impairment of the limbs below the injury site. SCI not only causes significant physical and psychological damage to patients but also places a heavy economic strain on society at large (1). In most countries, transport- and unintentional-related injuries are the primary causes of SCI (2). Armed conflicts in countries such as Saudi Arabia, Afghanistan, and Yemen have substantially contributed to increased years lived with disability (YLDs) (2, 3). After SCI, there are secondary injuries in addition to primary injuries (4). The primary injuries, encompassing ischemia, hypoxia, and oxidative stress, can precipitate further damage (5–7). In the secondary injuries, both the cells and the extracellular matrix (ECM) experience various alterations, ultimately leading to the creation of scar tissue that envelops the injury core and restricts axon regeneration, thus causing functional recovery to fail (8–10).

Following SCI, the secondary injury cascade rapidly drives the formation of two major types of pathological scars, namely glial scars and fibrotic scars, each composed of diverse and metabolically distinct cell populations whose behaviors are increasingly recognized to be governed by mitochondrial function (11). Glial scars, generated primarily by reactive astrocytes with contributions from microglia and oligodendrocyte precursor cells (OPCs), initially serve to contain inflammation and stabilize the lesion border, but can later develop into a biochemical barrier enriched in chondroitin sulfate proteoglycans (CSPGs) that restrict axonal regeneration (12, 13). Meanwhile, the fibrotic scar is formed by infiltrating fibroblasts, pericytes, endothelial cells, and immune-derived fibroblast-like cells, which deposit dense extracellular matrix (ECM) components such as collagen I/III and fibronectin. In the early phase, this fibrotic scaffold helps seal the necrotic core, stabilize tissue architecture, and prevent the spread of inflammation; however, as the scar matures, the excessively compact ECM within the lesion core forms a rigid physical barrier that markedly hinders axonal regrowth (14, 15).

Mitochondria, serving as the central hub for cellular energy metabolism, play a pivotal role in maintaining ATP synthesis, regulating redox homeostasis, and stabilizing calcium ion balance (16, 17). Emerging evidence now reveals that the balance between the adaptive and maladaptive roles of both scar types is tightly dictated by mitochondrial homeostasis within their constituent cells. Mitochondrial ATP depletion, excessive production of mitochondrial reactive oxygen species (mtROS), calcium overload, disrupted fusion–fission dynamics, and impaired mitophagy collectively reprogram astrocytes toward hypertrophic, CSPG-secreting phenotypes; drive microglia toward pro-inflammatory states; and enhance fibroblast myofibroblastic transformation and ECM overproduction (18–21). Conversely, maintenance of mitochondrial integrity supports astrocytic neuroprotection, modulates microglial polarization, preserves vascular stability, and limits excessive fibrosis (22). Thus, mitochondrial dysfunction acts as a central regulatory hub that not only shapes the cellular architecture of glial and fibrotic scars but also determines whether these scars ultimately stabilize the injured tissue or impose potent inhibitory barriers to neural repair.

**Figure 1:**
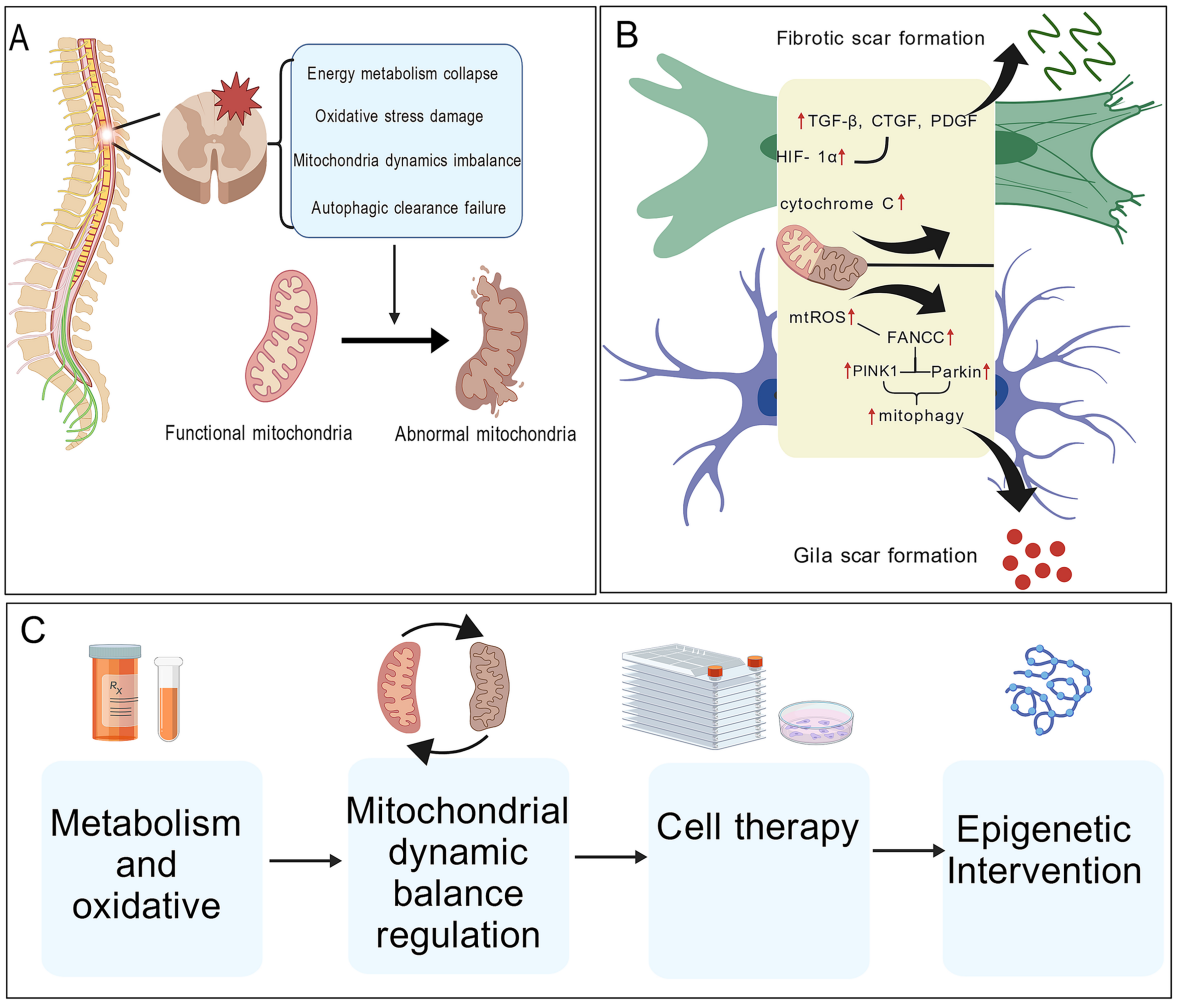
Effect of mitochondrial dysfunction on scar formation after spinal cord injury. **(A)** Spinal cord injury induces mitochondrial dysfunction characterized by energy metabolism collapse, oxidative stress, imbalance of mitochondrial dynamics, and impaired autophagic clearance, leading to the transition from functional to damaged mitochondria. **(B)** Mitochondrial dysfunction promotes fibrotic and glial scar formation through excessive mitochondrial ROS production, cytochrome c release, HIF-1α stabilization, and activation of profibrotic signaling pathways (e.g., TGF-β, CTGF, PDGF), as well as dysregulated PINK1/Parkin-mediated mitophagy in glial cells. **(C)** Therapeutic strategies targeting mitochondrial metabolism and oxidative stress, mitochondrial dynamic balance, cell-based therapies, and epigenetic interventions aim to restore mitochondrial homeostasis, attenuate pathological scarring, and promote neural repair after spinal cord injury. Created with BioGDP.com.

A graphical summary of this review is provided in Figure 1.

## 2 The impact of mitochondrial dysfunction on scar formation

Historically, mitochondrial research in SCI has focused predominantly on neurons. However, emerging scRNA-seq (Single-cell RNA sequencing) and metabolic profiling studies reveal that mitochondrial dysfunction in non-neuronal supporting cells, including astrocytes, OPCs, microglia, pericytes, and fibroblast, is equally critical for determining scar architecture and neuronal regenerative capacity (23). The formation of pathological scarring following SCI constitutes a complex pathological process, in which mitochondrial dysfunction plays a central driving role. This pathogenic mechanism involves the individual or synergistic effects of energy metabolism disorders, exacerbated oxidative stress, imbalance in mitochondrial dynamics, and dysregulation of the autophagy system (24–27). Notably, there exists a significant mechanistic divergence in how mitochondrial dysfunction influences glial scar formation versus fibrotic scar development (26, 28). This differential regulatory mechanism provides a critical theoretical foundation for targeted therapeutic interventions.

### 2.1 The impact of mitochondria on the formation of glial scars

Following spinal cord injury (SCI), the glial scar develops as a dynamic and stage-dependent structure whose function is tightly modulated by the mitochondrial status of its constituent cells, including astrocytes, microglia, and oligodendrocyte precursor cells (OPCs). The glial scar plays a dual role in the pathological process after SCI and other CNS injuries: both protective and inhibitory (12, 13). Recent studies have shown that the dysregulation of mitochondrial autophagy after SCI is a significant cause of excessive gliotic scar formation (18, 29).

In the early phase of injury, preserved mitochondrial homeostasis supports a predominantly protective glial response. In the research of Kandemir, they revealed during the acute injury phase, mechanical shock directly causes mitochondrial membrane rupture, releasing a large amount of mtROS and cytochrome c, activating Caspase-3-dependent astrocyte apoptosis (30). Recent studies have demonstrated that mitochondrial dysfunction following SCI can trigger astrocytic cell death, which in turn markedly exacerbates neurological deficits. Mitochondrial engulfment (mitophagy) plays a critical role in removing damaged mitochondria and preventing necroptotic spreading within the injured tissue. Overexpression of FANCC has been shown to enhance the expression of PTEN-induced kinase 1 (PINK1) and Parkin, thereby activating mitophagy, reducing astrocyte death, and ultimately supporting axonal regeneration and functional recovery (31). Balanced mitochondrial fusion–fission dynamics further sustain astrocyte survival and limit the release of inflammatory mediators. These mitochondrial-supported mechanisms facilitate the formation of a provisional glial barrier that stabilizes the lesion environment and restricts inflammatory spread.

However, as injury progresses toward the subacute and chronic phases, mitochondrial dysfunction gradually reprograms glial cells toward maladaptive and inhibitory states. Niu reported that activation of the JAK2/STAT3 pathway can enhance the migration of injured astrocytes and the overexpression of GFAP and scar tissue solidification (32). Xia revealed that mitochondrial calcium overload activates Calpain, promoting the release of IL-1β from the inflammatory body NLRP3, exacerbating scar formation (33). Excessive mtROS accumulation, calcium dysregulation, impaired mitophagy, and fragmented mitochondrial networks transform astrocytes into hypertrophic, CSPG-producing phenotypes that contribute to the biochemical components of the inhibitory scar.

Thus, mitochondrial regulation serves as a critical switch determining whether the glial scar adopts a beneficial, stabilizing role in the acute phase or evolves into a chronic inhibitory structure. Understanding this dual influence provides a mechanistic basis for therapeutic strategies aimed at enhancing mitochondrial integrity to preserve early protective functions while preventing the late-stage transition into excessive gliosis and biochemical inhibition.

### 2.2 The impact of mitochondria on the formation of fibrotic scar

Mitochondrial dysfunction can interfere with the normal functioning of endothelial cells, leading to increased vascular permeability, causing substances such as plasma proteins and inflammatory cells to seep out, triggering inflammatory responses and fibrosis. With the excessive deposition of fibrotic matrix, the hyperplasia of fibrous scar tissue occurs, ultimately resulting in organ dysfunction (26). Mitochondrial dysfunction leads to a reduction in ATP production and the accumulation of reactive oxygen species (ROS), which can cause tissue hypoxia and oxidative stress, thereby directly stabilizing the HIF-1α protein (inhibiting its ubiquitination and degradation) (34). The activated HIF-1α transcription factor upregulates profibrotic factors (such as TGF-*β*, CTGF, PDGF), stimulates the transformation of fibroblasts into myofibroblasts, and enhances the synthesis of extracellular matrix (ECM) components such as collagen I/III and fibronectin (35). Notably, similar mitochondrial ROS–HIF-1α–TGF-β signaling axes have been described in non-CNS chronic inflammatory and fibrotic disorders, suggesting that SCI-associated scarring shares mechanistic overlap with systemic fibrotic disease (36, 37).

In addition to fibroblasts and pericyte-derived fibroblast-lineage cells, macrophages accumulating in the lesion core also play a pivotal role in driving fibrotic scar formation after SCI. Ge et al. reported that exosomal miR-155 from M1-polarized macrophages promotes EndoMT(Endothelial-to-Mesenchymal Transition) and impairs mitochondrial function via activation of the NF-κB signaling pathway in vascular endothelial cells after SCI (38). Xu engineered a mitochondria-based therapeutic compound designed to selectively target macrophages at the injury site. Mitochondria were isolated from IL-10–induced, Mertk^hi bone marrow–derived macrophages and conjugated with the CAQK peptide (cationic–cysteine–alanine–glutamine–lysine), thereby enhancing their affinity for the damaged spinal cord microenvironment (39). This engineered mitochondrial compound markedly boosted macrophage-mediated phagocytosis of myelin debris, reduced lipid accumulation, restored mitochondrial function, and reduced fibrotic scar formation in both *in vitro* and *in vivo* models (39).

## 3 The functional conflicts of mitochondrial dysfunction in scar formation

Mitochondrial dysfunction participates in the complex, paradoxical relationship between the construction of physical barriers and the inhibition of axonal regeneration in the formation of pathological scars after spinal cord injury, through a dual mechanism. The core of this lies in the imbalance between the scar’s “protective isolation” and “regeneration obstruction” functions.

### 3.1 Early phase: metabolic stress as a trigger of protective barrier formation

The formation of a physical barrier after spinal cord injury initially serves a protective role by limiting the spread of inflammation and preventing secondary tissue damage. In the acute phase of SCI, mitochondrial dysfunction manifests as transient ATP deficiency and elevated reactive oxygen species (ROS) production. Moderate ROS levels activate redox-sensitive signaling pathways such as STAT3 in astrocytes, promoting their proliferation and hypertrophy. This process facilitates the rapid formation of a glial boundary that isolates necrotic tissue and restricts the spread of inflammatory mediators (40–43). Simultaneously, mitochondrial ROS activate the TGF-β1/Smad2 pathway in perivascular fibroblasts, driving their differentiation into myofibroblasts and initiating extracellular matrix deposition. The resulting glial–fibrotic interface acts as a structural barrier that stabilizes the lesion core. In this early context, mitochondrial stress contributes to injury containment and limits secondary injury (44, 45).

### 3.2 Chronic phase: persistent mitochondrial failure drives regenerative blockade

However, when mitochondrial dysfunction becomes sustained, the same bioenergetic stress transitions from adaptive to pathological.

**Figure 2:**
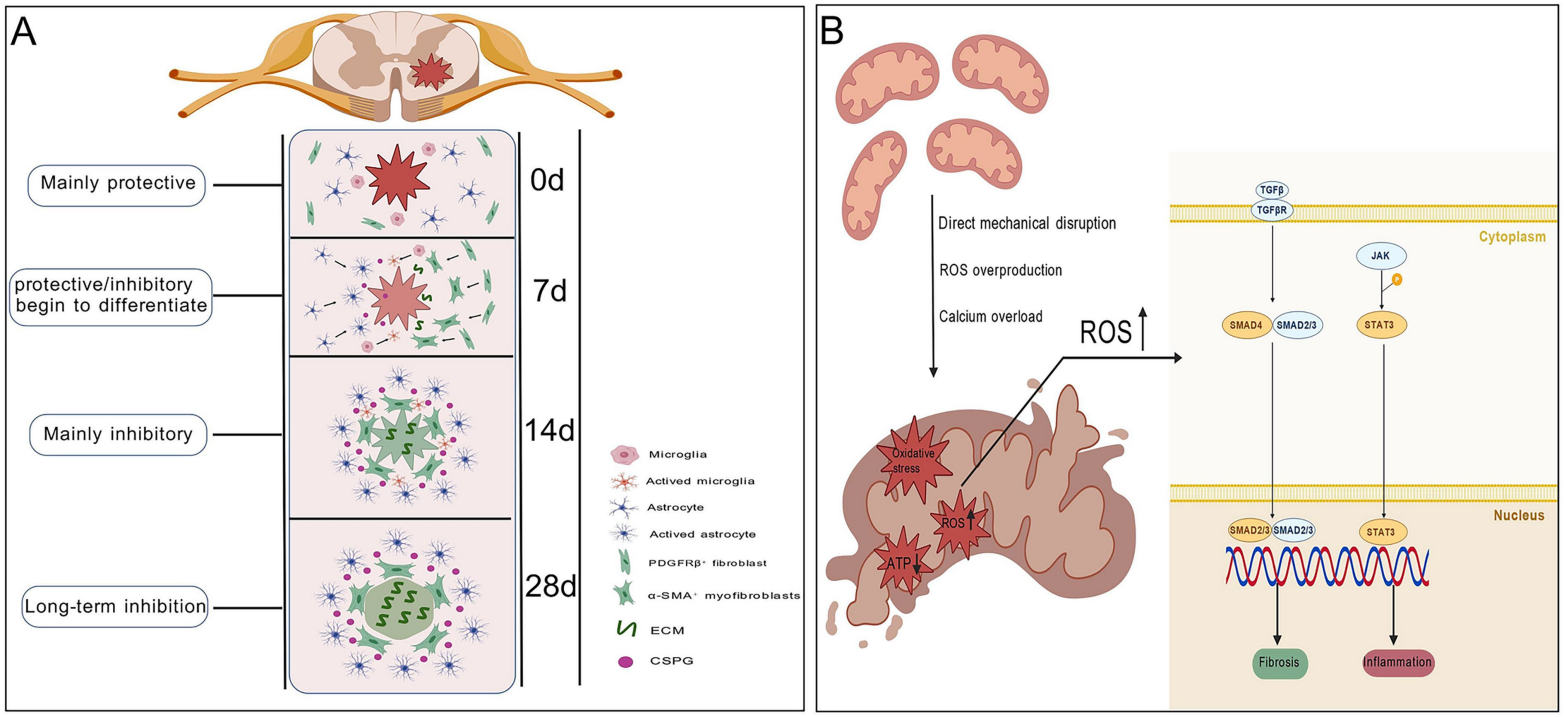
Temporal evolution of scar formation and mitochondrial ROS–mediated signaling after spinal cord injury. **(A)** Dynamic progression of glial and fibrotic scar formation after spinal cord injury, showing a transition from an early predominantly protective response (0 d) to progressively inhibitory and chronic scar structures at 7, 14, and 28 days. **(B)** Mitochondrial dysfunction induced by mechanical injury, oxidative stress, and calcium overload leads to excessive ROS production, activating TGF-β/SMAD and JAK/STAT3 signaling pathways to drive fibrosis and inflammation. Created with BioGDP.com.

Impaired mitophagy leads to accumulation of damaged mitochondria and persistent release of mitochondrial DNA and excessive ROS. These mitochondrial-derived danger signals activate the NLRP3 inflammasome in microglia, maintaining chronic sterile inflammation. The concept that mitochondrial damage-associated molecular patterns (mtDAMPs) sustain sterile inflammation has been extensively discussed in chronic musculoskeletal and inflammatory disorders. For instance, Wu et al. (46) summarized how impaired mitophagy and mtROS-driven inflammasome activation perpetuate fibrosis across tissues. These parallels reinforce the view that SCI scarring is not an isolated phenomenon but reflects conserved mitochondrial-inflammatory circuits observed across chronic fibrotic disorders (46). Pro-inflammatory cytokines further stimulate astrocytes to secrete axon growth–inhibitory molecules, including CSPGs, Ephrin-B2, and Semaphorin 3A, reinforcing a non-permissive biochemical environment (47–49). Meanwhile, in regenerating neurons, chronic mitochondrial fragmentation, often mediated by Drp1 overactivation, disrupts mitochondrial transport and local ATP supply within axons. Calcium overload and energy deficiency lead to growth cone collapse—growth cones are dynamic, actin-rich structures at axon tips that sense guidance cues and direct neurite outgrowth—thereby directly impairing axonal extension (50). Thus, mitochondrial dysfunction not only sustains the biochemical inhibitory milieu but also compromises the intrinsic regenerative capacity of neurons (51–53). Following spinal cord injury, distinct cell populations exhibit dynamic temporal and spatial changes that collectively shape the evolution of the lesion microenvironment. These spatiotemporal cellular dynamics, encompassing early inflammatory responses, progressive glial and fibrotic scar formation, and subsequent tissue remodeling, are summarized in Figure 2.

### 3.3 The temporal switch: from adaptive isolation to maladaptive rigidity

The paradox of “bidirectional inhibition” therefore lies not in simultaneous opposing effects, but in a temporal transition. Acute mitochondrial stress promotes scar formation as a protective barrier; chronic mitochondrial failure converts this barrier into a rigid, metabolically hostile structure that resists remodeling. This phase-dependent transformation suggests that therapeutic strategies targeting mitochondrial function must consider timing: early modulation may preserve protective containment, whereas later intervention should aim to restore mitophagy, rebalance redox signaling, and re-establish neuronal bioenergetic competence.

### 3.4 Reverse feedback: how scar microenvironment exacerbates neurodegeneration

Beyond its role as a structural and biochemical barrier, the mature scar microenvironment may actively accelerate neurodegeneration. Dense extracellular matrix deposition increases tissue stiffness, alters mechanotransduction signaling, and impairs nutrient diffusion within the lesion core (13, 15). Persistent inflammatory signaling from reactive astrocytes and microglia sustains elevated levels of cytokines and mitochondrial stressors, which further disrupt neuronal mitochondrial dynamics and bioenergetics. Moreover, hypoxic and metabolically restricted conditions within the compact fibrotic core may promote chronic mitochondrial fragmentation and impaired axonal transport, reinforcing neuronal vulnerability (4). Thus, scar formation and mitochondrial dysfunction may constitute a self-amplifying pathological loop, in which mitochondrial damage promotes scar maturation, and scar rigidity in turn exacerbates neuronal metabolic failure.

## 4 Treatment strategies for mitochondrial dysfunction

**Figure 3:**
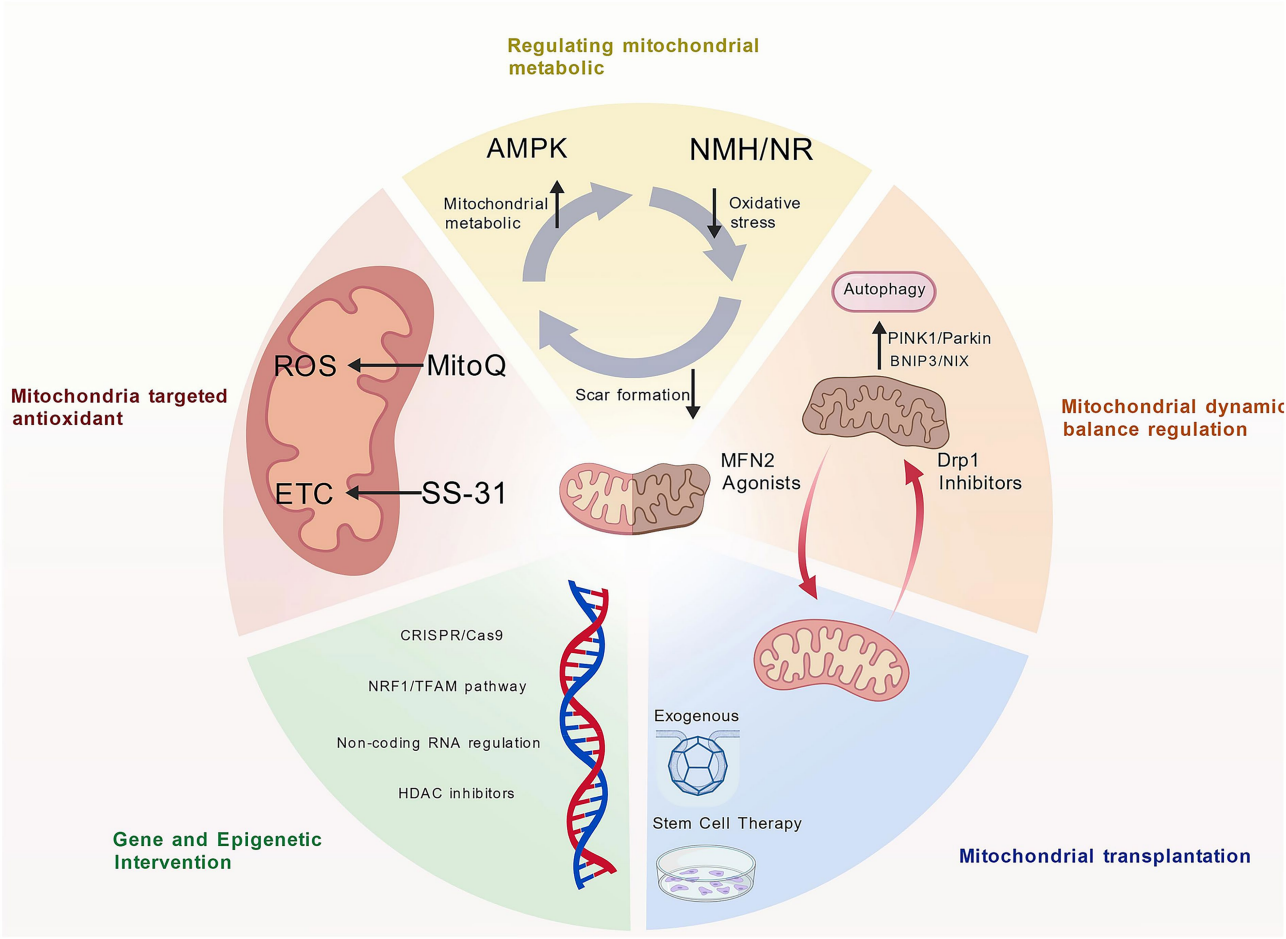
Therapeutic strategies targeting scar formation and neural repair. Various therapeutic strategies have been developed to modulate mitochondrial function following spinal cord injury (SCI), thereby suppressing scar formation, facilitating axonal regeneration, and promoting functional recovery. Created with BioGDP.com.

Mitochondrial dysfunction following spinal cord injury (SCI) is one of the core mechanisms leading to secondary damage and failure of neural repair. Processes such as collapse of mitochondrial energy metabolism, oxidative stress, dynamic imbalance, and autophagy defects exacerbate neuronal death, glial scar formation, and axonal regeneration inhibition. In recent years, therapeutic strategies targeting mitochondrial dysfunction have made significant progress in both basic and clinical research. Although many mitochondrial-targeted therapies primarily aim to restore bioenergetic homeostasis, their effects extend beyond neuronal survival. By reducing mtROS-driven STAT3 activation in astrocytes, suppressing TGF-β–mediated fibroblast-to-myofibroblast transition, and attenuating inflammasome activation in microglia/macrophages, these interventions indirectly but significantly modulate glial and fibrotic scar architecture. Current therapeutic strategies targeting scar formation and neural repair after spinal cord injury are summarized in Figure 3.

### 4.1 Mitochondrial metabolism and oxidative stress

#### 4.1.1 Mitochondria-targeted antioxidants

MitoQ: MitoQ is composed of the active portion of coenzyme Q10 (CoQ10) - the quinone ring, covalently bound to a lipophilic triphenylphosphonium (TPP) cation. Through the modification of TPP, MitoQ overcomes the challenge of CoQ10’s hydrophobicity, which makes it difficult to penetrate the mitochondrial membrane. This unique structure enables MitoQ to efficiently cross the double membrane of mitochondria and act directly on the inner mitochondrial membrane, thus achieving its targeted function (54). MitoQ primarily acts as an electron carrier; it can scavenge ROS, maintain membrane potential, protect mitochondrial DNA, and may involve anti-inflammatory and anti-apoptotic effects (55). Huang reported that MitoQ promotes functional recovery by enhancing angiogenesis and improving mitochondrial function after SCI (54).

SS-31 Peptide: SS-31 is a mitochondria-targeting tetrapeptide (D-Arg-Dmt-Lys-Phe-NH2) that exerts its protective effects by binding to cardiolipin within the inner mitochondrial membrane. Cardiolipin is a key phospholipid of the electron transport chain (ETC) complexes, but it is prone to oxidation and disruption of the mitochondrial membrane structure under pathological conditions. SS-31 stabilizes the conformation of cardiolipin, reducing the abnormal generation of reactive oxygen species (ROS), maintaining the mitochondrial membrane potential (ΔΨm), thereby improving ATP synthesis efficiency and inhibiting the opening of the mitochondrial permeability transition pore (mPTP). Additionally, SS-31 can block the release of cytochrome C, alleviating oxidative stress-induced apoptosis (56). In spinal cord injury models, SS-31 significantly reduces astrocyte activation and glial scar formation, and promotes axonal regeneration (57, 58).

#### 4.1.2 Regulating mitochondrial metabolic pathways

Activation of the AMPK Pathway: AMPK (AMP-activated protein kinase) activation promotes neural repair through a dual mechanism following spinal cord injury (SCI). Firstly, AMPK enhances mitochondrial biogenesis (activating the PGC-1α pathway) and autophagy, improving neuronal energy metabolism, reducing oxidative stress and mitochondrial dysfunction, thereby inhibiting Caspase-3-mediated neuronal apoptosis (59–63). Secondly, AMPK suppresses the expression of pro-inflammatory factors (such as IL-6) and inhibitory matrix molecules (such as CSPGs) by inhibiting NF-κB and STAT3 signaling pathways in astrocytes, limiting excessive gliosis (62). Research has shown that the AMPK agonist AICAR significantly reduces scar area in SCI model mice and promoted the recovery of motor function, confirming its coordination of neuroprotection and microenvironment regulation through metabolic reprogramming (64).

Supplementation with NAD + precursors (such as NMN and NR) increases intracellular NAD + levels, activates SIRT1, enhances mitochondrial energy metabolism, and inhibits oxidative stress, blocking the Caspase-3 dependent apoptotic pathway, thereby reducing neuronal apoptosis after spinal cord injury (SCI) (65, 66). At the same time, NAD + inhibits the over-activation of astrocytes and the secretion of chondroitin sulfate proteoglycans (CSPGs) by suppressing NF-κB and STAT3 signaling, inhibiting the formation of glial scars (67).

### 4.2 Mitochondrial dynamic balance regulation

#### 4.2.1 Inhibit excessive mitochondrial fission

Drp1 Inhibitors: Drp1 (Dynamin-related protein 1) is a key protein involved in mitochondrial fission. Its overactivation leads to excessive mitochondrial division after SCI, resulting in mitochondrial fragmentation in astrocytes, ROS burst, and apoptotic signaling. Inhibiting Drp1 (such as with Mdivi-1) can stabilize the mitochondrial network, reduce mtROS release, block NLRP3 inflammasome activation, and IL-1β secretion, thereby inhibiting excessive proliferation of astrocytes and scar formation (49, 68). Song’s research confirmed that Drp1 inhibitor treatment can prevent mitochondrial fission, inhibit pro-inflammatory microglial polarization, significantly reduce the glial scar area in SCI mouse models, promote neural recovery from spinal cord injury, and improve motor function. Therefore, targeting the DRP1 axis represents a new approach to treating SCI (48).

MFN2 Agonists: MFN2 (mitofusin 2) agonists enhance mitochondrial fusion, repair the mitochondrial network disrupted after damage, and boost energy metabolism and calcium homeostasis. MFN2 agonists, such as MASM7(mitofusin activating small molecule7)can activate MFN2-GTPase activity, reduce mitochondrial fragmentation, inhibit astrocyte apoptosis, and suppress the release of pro-inflammatory factors (such as TNF-*α*) (69–71).

#### 4.2.2 Enhance mitochondrial autophagy

Activation of the PINK1/Parkin Pathway: Following SCI, due to the dysfunction of mitochondrial autophagy (mitophagy), damaged mitochondria cannot be effectively cleared, leading to their accumulation and exacerbating oxidative stress and neuroinflammation. The PINK1/Parkin pathway constitutes a key mechanism for mitochondrial quality control: when the mitochondrial membrane potential decreases, PINK1 stably localizes on the outer mitochondrial membrane and recruits and activates the E3 ubiquitin ligase Parkin. Subsequently, Parkin ubiquitinates mitochondrial proteins, marking these damaged mitochondria for recognition and engulfment by autophagosomes (72–75).

Targeting the BNIP3/NIX Pathway: The BNIP3/NIX pathway is a key regulator of mitochondrial autophagy (mitophagy), which clears damaged mitochondria through a pathway independent of PINK1/Parkin. The activation of the BNIP3/NIX pathway via gene editing or drugs can enhance mitochondrial quality control. After spinal cord injury, the downregulation of BNIP3 expression in astrocytes leads to defects in mitochondrial autophagy, causing the accumulation of mtROS and activation of TGF-β1 signaling, which drives glial scar hardening. Overexpression of BNIP3 specifically enhances mitochondrial clearance efficiency, reduces the release of apoptotic bodies, and inhibits the expression of fibrosis-related genes (such as COL1A1, *α*-SMA) (76–78). The study of Yao confirmed the effects of LIG-enhanced mitophagy through BNIP3-LC3, providing new therapeutic targets and strategies for repairing SCI (79).

### 4.3 Mitochondrial transplantation and cell therapy

#### 4.3.1 Exogenous mitochondrial transplantation

Direct mitochondrial delivery: Healthy mitochondria isolated through intravenous or local injection are transplanted to the damaged area to repair cellular energy metabolism defects. A research of Raciti indicates that intranasal delivery of exogenous mitochondria can cross the blood–brain barrier, be taken up by astrocytes and neurons in the spinal cord injury area, significantly restoring ATP levels and reducing fibrotic scarring (80).

Nanocarrier-mediated delivery: Liposomes or polymer nanoparticles encapsulating mitochondria represent a technique that can significantly improve targeted delivery efficiencyof mitochondria and their survival rate within organisms. MITO-Porter is a special nanodelivery carrier capable of effectively delivering functional mitochondria to specific cells (81, 82). In a study on a mouse model of spinal cord injury (SCI), researchers successfully utilized the MITO-Porter system to deliver functional mitochondria to damaged neurons, thereby demonstrating the great potential of this technology in treating neurological diseases (83).

#### 4.3.2 Stem cell therapy

Mesenchymal Stem Cell (MSCs) Exosomes: MSCs carry functional mitochondrial components (such as mtDNA, ATP synthase subunits, and metabolic enzymes), which are directly integrated into the host cell mitochondria through intercellular transfer, repairing oxidative phosphorylation defects (84–87). A 2023 study published in “Advanced Science” showed that MSCs exosomes can upregulate the expression of PGC-1α in host cells, promoting mitochondrial biogenesis, and by delivering antioxidant proteins (such as SOD2) clear ROS, restoring mitochondrial membrane potential (88). Exosome treatment significantly reduced mitochondrial fragmentation in astrocytes, inhibited the proliferation of glial scars, and improved neuronal survival rates (axon regeneration increased by 30%) (89).

Mitochondria Derived from Induced Pluripotent Stem Cells (iPSCs): iPSCs exhibit high metabolic activity, with significantly higher expression of electron transport chain complexes than mature cells, enabling efficient ATP production and reducing the accumulation of ROS (90–92). After transplantation of iPSC mitochondria into neurons, oxidative damage is reduced by more than 50% through enhanced glutathione peroxidase (GPx) activity and upregulation of SOD2 expression, while inhibiting mitochondria-dependent apoptotic pathways (such as Caspase-3) (93). Furthermore, the unique metabolic reprogramming ability of iPSCs can promote synaptic reconstruction in damaged neurons and decrease the area of glial scars (94).

### 4.4 Gene and epigenetic intervention

#### 4.4.1 Mitochondrial gene editing

CRISPR/Cas9 targeting of mtDNA: Due to the presence of the mitochondrial membrane and the lack of effective delivery methods, the traditional CRISPR/Cas9 system has encountered difficulties in editing mitochondrial DNA (95). New targeting mtDNA technologies such as DdCBE, TALED may break through the limitations of the traditional CRISPR system, targeting and repairing mtDNA mutations (96–98). Cho’s report showed that DdCBE (double-stranded DNA deaminase) delivered via AAV successfully corrected ND4 mutations in a mouse model, restored complex I function, reduced neuronal mitochondrial ROS and apoptosis (97). DdCBE provides precise treatment possibilities for both hereditary and acquired mitochondrial diseases, but the efficiency of *in vivo* delivery and off-target risks still need optimization.

Nuclear gene regulation: Overexpression of PGC-1α (peroxisome proliferator-activated receptor gamma coactivator 1-alpha) can activate the NRF1/TFAM pathway, driving the expression of genes related to mitochondrial biogenesis (such as COX5B, ATP5F1), and enhancing mitochondrial DNA (mtDNA) replication and respiratory chain complex assembly (99–101). Studies have shown that after spinal cord injury, AAV-mediated overexpression of PGC-1α significantly increases neuronal ATP levels, reduces mitochondrial oxidative damage, and by inhibiting the transformation of astrocytes into a pro-fibrotic phenotype, reduces the area of scarring. This strategy provides a new direction for targeting nuclear-mitochondrial interactions to improve metabolic imbalance after SCI (63).

#### 4.4.2 Epigenetic modification

Histone deacetylase (HDAC) inhibitors: HDAC inhibitors block the process of histone deacetylation, thereby increasing the acetylation level of chromatin, which in turn promotes the expression of mitochondria-related genes (102, 103). VPA can enhance mitochondrial antioxidant defense by increasing the level of SOD2 (superoxide dismutase 2), reducing the accumulation of ROS (reactive oxygen species); in addition, VPA can activate CPT1A (carnitine palmitoyltransferase 1A), thereby promoting the oxidation process of fatty acids and optimizing energy metabolism. Although not directly investigated in SCI models, these mitochondrial regulatory mechanisms may provide mechanistic insights relevant to post-injury metabolic dysregulation (104, 105).

Non-coding RNA regulation: Non-coding RNAs may regulate gene expression by targeting specific mRNAs. miR-338 inhibits the translation of the mRNA for cytochrome C oxidase subunit COX4I1, thereby modulating mitochondrial respiratory chain function and reducing neuronal apoptosis (106–108).

Several therapeutic strategies targeting scar formation and neural repair after spinal cord injury have been developed. These approaches, targeting distinct cellular populations and molecular pathways, are summarized and presented in detail in Table 1.

**Table 1:** Mitochondria-targeted therapeutic strategies for spinal cord injury.

Therapeutic agent/intervention	Molecular target/mechanism of action	Primary target cell type(s)	Functional effects in SCI
Mitochondrial metabolism and oxidative stress			
MitoQ	Mitochondria-targeted antioxidant; scavenges mtROS; stabilizes membrane potential	Astrocytes Microglia Neurons	Reduces oxidative stress and inflammation
SS-31 (Elamipretide)	Binds cardiolipin in the inner mitochondrial membrane; protects ETC activity	Neurons Astrocytes	Preserves mitochondrial function; reduces apoptosis
AMPK activators	Activate AMPK → enhance mitochondrial biogenesis and metabolic regulation	Astrocytes Microglia Neurons	Improves energy metabolism; reduces inflammatory signaling
Mitochondrial dynamic balance regulation			
Mdivi-1 (Drp1 inhibitor)	Inhibits Drp1-mediated mitochondrial fission	Astrocytes Microglia	Reduces mitochondrial fragmentation
MFN2 agonists / overexpression	Promotes mitochondrial fusion; restores mitochondrial dynamics	Neurons Astrocytes OPCs	Enhances energy homeostasis
Mitochondrial transplantation and cell therapy			
Mitochondrial transplantation	Transfers functional exogenous mitochondria into injured cells	Neurons Astrocytes Microglia	Alleviates energy crisis; increases cell survival; promotes tissue repair
MITO-Porter nanocarriers	Nanocarrier-mediated targeted mitochondrial delivery	Neurons Glial cells	Enhances mitochondrial uptake; restores energy metabolism
Stem cell–derived extracellular vesicles (mito-EVs)	Transfer mitochondrial proteins, mtDNA, and metabolic regulators via vesicles	Neurons Astrocytes Microglia	Rebuilds mitochondrial function; reduces inflammation; supports regeneration
iPSC-derived mitochondrial transfer	Functional mitochondria transferred from pluripotent stem cells	Neurons, Glial cells	Enhances metabolic recovery; protects against degeneration; limits scarring
Gene and epigenetic intervention			
PINK1/Parkin pathway activators	Enhance mitophagy to eliminate damaged mitochondria	Astrocytes, Microglia	Decrease mtROS; suppress inflammasome activation
BNIP3/NIX modulators	Regulate receptor-mediated mitophagy	Astrocytes, macrophages, fibroblasts	Reduce mitochondrial stress
HDAC inhibitors	Modify metabolic gene expression; regulate mitochondrial dynamics	Astrocytes Fibroblasts	Reduce pro-fibrotic and pro-inflammatory signaling
miRNAs (e.g., miR-21, miR-146a)	Regulate mitochondrial biogenesis and mitophagy-related genes	Astrocytes Microglia	Modulate inflammation; improve mitochondrial metabolism; promote repair

Summary of representative interventions targeting mitochondrial metabolism, dynamics, mitophagy, and mitochondrial transfer, along with their molecular mechanisms, primary target cell types, and reported effects on scar formation and neural repair after spinal cord injury.

### 4.5 Translational limitations of mitochondrial-targeted therapies

Despite promising preclinical results, several barriers hinder clinical translation of mitochondrial-targeted therapies in SCI. First, mitochondrial dysfunction is highly cell-type specific, yet most pharmacological interventions lack targeting precision, potentially disrupting beneficial acute-phase responses. Second, mitochondrial transplantation strategies face immunogenicity concerns and limited long-term engraftment efficiency. Third, modulation of mitophagy or fusion–fission balance may exert opposing effects depending on injury stage. Finally, rodent models fail to replicate the structural complexity and chronic inflammation observed in human SCI, contributing to translational failure. Future therapeutic strategies must incorporate spatiotemporal precision and combinatorial approaches rather than single-target antioxidant interventions.

## 5 Challenges and future directions

### 5.1 Mechanistic challenges

Although major advances have been made in understanding mitochondrial dysfunction in scar formation, several critical gaps remain. First, the heterogeneous cellular composition of the SCI microenvironment complicates mechanistic dissection. Astrocytes, microglia, OPCs, pericytes, fibroblasts, and infiltrating macrophages each exhibit unique mitochondrial metabolic profiles and stress responses, leading to cell-type–specific contributions to glial versus fibrotic scarring. Second, mitochondrial signaling exhibits marked temporal heterogeneity, being protective in the acute phase but inhibitory in the chronic phase, which complicates the development of stage-specific interventions. Third, intercellular communication mediated by mitochondrial signals, including mtROS, mtDNA, mitophagy-derived vesicles, and metabolic coupling, is insufficiently mapped. How mitochondrial dysfunction in astrocytes influences fibroblast activation, or how macrophage mitochondria regulate glial phenotype, remains poorly understood. Additionally, current methodologies lack the ability to visualize mitochondrial dynamics *in vivo* with single-cell spatial resolution, and rodent models fail to fully replicate human scar complexity.

### 5.2 Mechanistic future directions

Future research must aim to construct a multi-layered atlas of mitochondrial regulation in SCI. This includes integrating spatial transcriptomics, single-cell metabolomics, and real-time mitochondrial imaging to precisely track how mitochondrial pathways evolve in specific cell types. Elucidating mitochondria-centered cell–cell communication networks, including astrocyte–microglia metabolic coupling, macrophage-sourced mitochondrial stress signals driving fibroblast activation, will be key to understanding scar architecture. Importantly, identifying mitochondrial tipping points between protective and inhibitory states may reveal optimal therapeutic windows. Human stem cell–derived organoids and 3D SCI models offer promising platforms to bridge translational gaps and improve mechanistic relevance.

### 5.3 Therapeutic challenges

Despite significant progress, several limitations restrict translation of mitochondrial-targeted therapies. Achieving cell-specific mitochondrial delivery remains difficult: astrocytes, fibroblasts, and macrophages differ in uptake capacity and mitochondrial dynamics. Mitochondrial transplantation faces concerns regarding immune activation, long-term survival of transplanted mitochondria, and insufficient targeting precision. Gene-editing approaches (TALED, DdCBE) encounter barriers related to delivery efficiency and off-target risks. Moreover, the dual roles of scars, protective early, inhibitory later, mean that mitochondrial modulation must be precisely timed; inappropriate intervention may disrupt beneficial acute responses or exacerbate chronic inhibition. Combination therapies remain underexplored, and integrating mitochondrial repair with ECM remodeling, neuroimmune modulation, and axon-intrinsic regeneration remains challenging.

### 5.4 Therapeutic future perspectives

Next,-generation therapies should emphasize spatiotemporal precision and cell-type specificity. Peptide-guided nanoparticles, mitochondria-targeting liposomes, and optogenetic mitochondrial controllers enable more accurate modulation of metabolic and inflammatory pathways. Engineering mitochondria, through protein modification, genetic enhancement of fusion/mitophagy pathways, or immuno-compatible optimization, may greatly improve transplantation efficiency. Combined strategies integrating mitochondrial protection + scar remodeling (e.g., MitoQ + Chondroitinase ABC) hold potential for synergistic repair by simultaneously restoring metabolic homeostasis and reducing structural inhibition. Furthermore, coupling mitochondrial therapy with immunometabolic reprogramming of macrophages, biomaterial scaffolds, and axonal growth–promoting gene therapy may reshape the inhibitory microenvironment and enhance functional recovery.

## 6 Conclusion

Mitochondrial dysfunction is the core driver of scar formation following SCI, and targeting mitochondrial metabolism and dynamics provides a new direction for improving neural repair. The therapeutic strategies for mitochondrial disorders post-spinal cord injury are evolving from single antioxidant interventions to multimodal combined treatments. In the future, it will be necessary to combine gene editing, cell engineering, and materials science to develop new technologies for precise temporal and spatial regulation of mitochondrial function, while also promoting clinical trials to verify their safety and efficacy. All figures presented in this study were generated using the BioGDP platform (109).
